# Assessing sleep health dimensions in frontline registered nurses during the COVID-19 pandemic: implications for psychological health and wellbeing

**DOI:** 10.1093/sleepadvances/zpac046

**Published:** 2022-12-16

**Authors:** Allison A Norful, Fatemeh Haghighi, Ari Shechter

**Affiliations:** Columbia University School of Nursing, New York, NY, USA; Icahn School of Medicine at Mount Sinai, New York, NY, USA; U.S. Department of Veterans Affairs, James J Peters VA Medical Center, New York, NY, USA; Columbia University Irving Medical Center, New York, NY, USA

**Keywords:** sleep, nurses, burnout, anxiety, depression, COVID-19

## Abstract

The COVID-19 pandemic altered work environments of nurses, yielding high rates of stress and burnout. Potential protective factors, including effective sleep, may influence psychological health and wellbeing. Evidence about sleep in nurses may help develop interventions that mitigate burnout and poor psychological outcomes. A cross sectional survey was distributed across three hospitals to nurses in New York City (NYC). During the first wave of the pandemic (March–April 2020), NYC had the highest incidence of laboratory-confirmed COVID-19 cases (915/100 000) and half of all COVID-related deaths nationwide. Multivariable logistic regression was used to determine associations between Pittsburgh Sleep Quality Index (PSQI) global sleep score, PSQI sleep dimensions, and psychological health (burnout, depression, anxiety, and compassion fatigue), unadjusted and then controlling for individual and professional characteristics. More than half of the participants reported burnout (64%), depression, (67%), and anxiety (77%). Eighty percent of participants had PSQI global scores >5 (poor sleep) (mean 9.27, *SD* 4.14). Respondents reporting good sleep (PSQI ≤ 5) had over five times the odds of no burnout (OR: 5.65, 95% CI: 2.60, 12.27); increased odds of screening negative for depression (OR: 6.91, 95% CI: 3.24, 14.72), anxiety (OR: 10.75, 95% CI: 4.22, 27.42), and compassion fatigue (OR: 7.88, 95% CI: 1.97, 31.51). Poor subjective sleep quality PSQI subcomponent was associated with burnout (OR: 2.21, 95% CI: 1.41, 3.48) but sleep duration subcomponent was not (OR: 0.84, 95% CI: 0.59, 1.19). Daytime dysfunction was significantly associated with all psychological outcomes. Sleep disturbances and medications yielded higher anxiety odds. Overall, sleep quality appears more strongly related to burnout than sleep duration in nurses working during the COVID-19 pandemic. Sleep interventions should target individual sleep dimensions in nurses.

**Statement of Significance**

Burnout and poor psychological health risk in nurses have reached alarming proportions during the COVID-19 pandemic. This is among the first studies to investigate the influence of individual sleep health dimensions on the psychological health of nursing workforce. It appears that subjective sleep quality and not sleep duration is significantly associated to burnout. Further, daytime dysfunction appears to increase the odds of adverse psychological health outcomes including depression and anxiety. Sleep disturbances and the use of sleep medication also appear to increase the odds of anxiety. Effective sleep may serve as a protective factor against depression, anxiety, burnout, and compassion fatigue, while increasing the odds of resilience in nurses working on the frontlines of the COVID-19 pandemic.

## Introduction

Prolonged exposure to stressful environments and situations has been associated with multiple adverse psychological outcomes, and accumulating research has highlighted the psychological toll of engaging in clinical work [1, 2]. Even under routine conditions, irrespective of COVID-19 work environments, healthcare workers (HCWs), especially nurses, were at disproportionate risk for burnout, depression, anxiety, and suicidal behavior [3, 4]. Moreover, past studies have illuminated that HCWs who experience burnout are at increased risk for anxiety, depression_,_ and substance abuse [5, 6]. In addition to its essential role in the regulation of a variety of cardiometabolic processes [7], sleep is directly implicated in regulating mood states, and sleep disturbances are a hallmark of many psychological disorders, including anxiety, posttraumatic stress disorder, depression, and burnout [8]. In terms of emotional processing, disturbed sleep can lead to enhanced reactivity to negative emotions, such as anger and fear, and reduced reactivity to positive stimuli [9]. Sleep loss is also linked to reports of reductions in self-esteem, empathy toward others, interpersonal functioning, stress management, and coping skills [10].

Several challenges that HCWs routinely face were magnified by the COVID-19 pandemic. A previous study noted that HCWs (e.g. nurses, physicians, and nursing assistants respiratory therapists) reported unique challenges in care delivery including fears of COVID-19 exposure and threats to the safety of themselves and their loved ones [11]. Indeed, HCWs treating COVID-19 patients have experienced significant acute psychological symptoms [12]. As the pandemic response progressed, there was increased evidence that nurses were impacted psychologically at a significantly higher rate compared to other HCW disciplines [13]. In an early study investigating hospitals in and around Hubei province in China during the COVID-19 pandemic, over 70% of survey respondents reported distress, 45% reported anxiety, and 34% reported insomnia, with more severe symptoms found in nurses [14]. A second COVID-19 study explored unique nursing experiences during the pandemic and found that perceived personal safety risk due to higher patient exposure for nurses, while simultaneously having a “sense of duty,” contributed to increased stress and burnout [12]. The higher severity of psychological symptoms in nurses was also noted in a large cross sectional survey conducted during the peak of COVID-19 cases in New York City in April and May 2020. This study found that 64% of nurses screened positive for acute stress disorder (i.e. early PTSD) compared to 40% of attending physicians, 53% of nurses screened positive for depressive symptoms compared to 38% for physicians, and 40% of nurses screened positive for anxiety symptoms compared to 15% for physicians [13]. Sleep disturbances were common, with over 70% of HCWs in the study reporting insomnia symptoms of at least moderate severity, and severity of insomnia symptoms also differed by group, with nurses reporting worse sleep problems versus physicians. That study, however, was limited in its use of only a non-validated single-item question to assess sleep disturbance, and lack of assessment of other factors that are important for HCW well-being and function like burnout, compassion fatigue, and resilience (which may buffer against the adverse effects of subsequent life stressors). Collectively, previous evidence shows that the psychological health and wellbeing of nurses have been uniquely impacted by the pandemic compared to other HCW disciplines [15]. Additional studies have investigated sleep in nurses yet most studies were conducted pre-pandemic, thus reducing our understanding of how sleep health in nurses relates to psychological factors like burnout in light of the current pandemic work environments [16]. We subsequently evaluated the qualitative perspectives of frontline workforce in the northeast region of the United States and HCW including nurses reported sleep disturbances including insomnia [11]. Yet, it remained unclear if specific dimensions of sleep (e.g. latency, duration, and disturbances) were associated with psychological health risk, including burnout, depression, and anxiety. Given the enormity of the challenges that have plagued nurses’ mental health during the pandemic, an evaluation of sleep, and individual sleep dimensions is essential, as it can help inform practices to protect long-term well-being and functioning of this individual workforce group. Nurses make up a unique frontline HCW population with different approaches to care and increased direct patient contact compared to other disciplines, including higher exposure to death and dying and ethical challenges [17–19]. Subsequently, nursing-specific interventions that may mitigate burnout and other poor psychological health outcomes should be investigated. As sleep is a modifiable health behavior it may present a potential therapeutic target to improve other psychological outcomes in nurses. Effective sleep may serve as a health-promoting behavior to optimize mental health in nursing workforce. In this study, our goal was to examine multidimensional sleep characteristics in frontline nurses during the COVID-19 pandemic and the relationship with various psychological health measures. We hypothesized that good overall sleep quality (assessed with the Pittsburgh Sleep Quality Index [PSQI] global score) would be associated with reduced burnout (primary outcome), as well as reduced depression, anxiety, and compassion fatigue (secondary outcomes). We also hypothesized that higher sleep duration and lower sleep disturbances, as assessed by these PSQI sleep health dimensions scores, would be associated with decreased presence of the same adverse psychologic health outcomes.

## Methods

### Design, sample, and setting

Ethical approval was obtained from Columbia University Irving Medical Center Institutional Review Board. We conducted a cross sectional electronic survey of registered nurses across three hospitals in the New York City region; (1) Hospital A is an academic medical center with greater than >700 beds and 1000 employed nurses; (2) Hospital B is a 196-bed community hospital serving both an urban and suburban population with 200 nurses; (3) Hospital C is 128-bed community hospital with 200 nurses and serving a suburban and rural community. Potential participants were eligible if: (1) Currently employed as a registered nurse; (2) delivering patient care during the first wave of the COVID-19 pandemic in the New York City region; (3) have access to email; and (4) Read and understand English language. Nurses working in administrative roles, outpatient settings, or within an advanced practice role (e.g. Nurse practitioner) or non-nursing discipline were excluded. The three hospitals were purposively selected to represent care delivery in urban, suburban, and rural patient settings.

### Survey measures

Survey measures consisted of demographic questions and validated instruments to scale psychological outcomes described below. We test-administered the survey in a group of five people who completed all items in approximately 15 min and did not report survey fatigue.

*Demographics* were assessed with items that asked the participants to report their age, gender, race, ethnicity, highest degree of education, years of experience, number of years in primary position, and which unit/service line they primarily work within. *Burnout* and *Job Satisfaction were* measured using two validated items. The *Burnout* item asked the respondent to identify which response about burnout symptoms they identified most [20]. There were five response options: (1) I enjoy my work. I have no symptoms of burnout; (2) Occasionally, I am under stress, and I do not always have much energy as I once did, but I do not feel burned out; (3) I am definitely burning out and have one or more symptoms of burnout, such as physical and emotional exhaustion; (4) The symptoms of burnout that I am experiencing would not go away. I think about frustration at work a lot; (5) I feel completely burned out and often wonder if I can go on. I am at the point where I may need some changes or may need to seek some sort of help. We dichotomized the item responses combining the first two responses (little to no burnout) and the remaining three responses (demonstrates some level of burnout). The *Job Satisfaction* item asked, “On the whole how satisfied are you with your present job?” Responses included a four-point Likert scale ranging from “very satisfied” to “very dissatisfied.” We dichotomized the responses to “satisfaction” (first two responses) and “dissatisfaction” (last two responses).

*Depression* was screened using the Patient Health Questionnaire-2 (PHQ-2) [21]. The PHQ-2 asks about the frequency of depressed mood and anhedonia over the past 2 weeks. The two items included: Over the last 2 weeks, how often have you been bothered by the following problems; (1) little interest or pleasure in doing things; and (2) feeling down, depressed, over hopeless. Responses are in the form of a Likert scale from “not at all” (0) to “nearly every day” (3). The scores for both items are totaled. A score greater than or equal to 3 indicates the presence of depression and a score below 3 indicates the absence of a positive depression screening. PHQ-2 has a sensitivity of 82.9% and specificity of 90%.

*Anxiety* was screened using Generalized Anxiety Disorder-2 (GAD-2) [22], a two-item instrument that asks the respondent to report “over the last 2 weeks, how often have you been bothered by the following problems? (1) feeling nervous, anxious, or on edge; (2) not being able to stop or control worrying.” Responses range from “not at all” (0) to “nearly every day” (3). The GAD-2 score is obtained by adding the score of each question and a cutoff of 3 and/or above indicates the presence of anxiety while scores less than 3 indicate the absence of anxiety. GAD-2 has a sensitivity of 86% and specificity of 83%.

*Compassion fatigue* was measured using the Compassion Fatigue Self-test for Helpers [23], a 66-item instrument that asks participants to rate each item from “never” (0) to “very often” (5). The items include both personal attributes and items about being “a helper with a helping environment.” Of the 66 items, 23 items are specific to the construct of compassion fatigue. Total item scores were tallied to generate a compassion fatigue risk score (26 or less = extremely low risk, 27–30 = low risk; 31–35 = moderate risk; 36–40 = high risk; 41 or more = extremely high risk). We dichotomized the score at the level of 30, indicating little to no risk (<30) and moderate to extremely high risk (≥30).

*Resilience*, defined as a person’s coping ability, was scaled with the Brief Resilience Scale [24]. The possible score ranges from 1 (low resilience) to 5 (high resilience). Respondents are asked to rate each item on a five-point Likert scale from “strongly disagree” to “strongly agree.” Items such as “I have a hard time making it through stressful events” and “It is hard for me to snap back when something bad happens” are used. The response scores are added and divided by the number of items answered to get the final score. A score less than 3 indicates low resilience.

*Sleep* was assessed using the Pittsburgh Sleep Quality Index [25], a 19-item self-rated instrument, that measures seven sleep dimensions: subjective sleep quality; sleep latency; sleep duration, habitual sleep efficiency, sleep disturbances, use of sleep medication, and daytime dysfunction. Responses consist of both open text (e.g. bed time) and Likert scale (“not during the past month” to “three or more times a week”). The PSQI is the most widely used measure of global sleep quality with good reliability and validity. The process to calculate the global PSQI score is described in our data analysis section below.

### Data collection

The survey measures were uploaded into Qualtrics survey software, which generates an anonymous link embedded into the recruitment email. The email consisted of an explanation of the survey, anticipated completion time, its anonymous nature, contact information for the research team, and the link to access the survey directly. Due to the anonymity of the surveys, a waiver of written consent was obtained since this would be the only document linking the participants to their responses. Hospital-wide nursing email list serves were obtained from nursing leadership. The initial recruitment email was sent in October 2020 and an email reminder was sent after 2 weeks. The survey window was left open for 1 month after which the survey was deactivated and data were exported to a Microsoft Excel spreadsheet for cleaning and coding.

### Data analysis

The PSQI contains 19 items focusing on sleep duration, quality, time taken to fall asleep, and frequency and severity of several sleep-specific problems, that reflect usual sleep habits and experiences on the majority of days and nights over the prior month. These 19 items are grouped into the following seven categories, describing different sleep dimensions: subjective sleep quality, sleep latency (i.e. time taken to fall asleep), sleep duration, habitual sleep efficiency (i.e. amount of time spent sleeping relative to time spent in bed), sleep disturbance, use of sleep medication, and daytime dysfunction. Each of the seven sleep dimensions are scored on a 0–3 scale, with 0 indicating better and 3 indicating worse. The global PSQI score is calculated by summing the scores on each of the seven equally weighted components, and ranges from 0 to 21 with higher scores indicating worse overall sleep. A global PSQI score cutoff value of >5 is used to indicate poor sleep quality [24].

A final data set was imported into SPSS for data analysis. Outcome variables (burnout, depression, anxiety, compassion fatigue, job satisfaction, and resilience) were dichotomized as described under each measure above. Sample characteristics were analyzed and presented as mean and standard deviation for continuous variables (e.g. age, years of experience), and absolute and relative frequencies (%) for categorical variables (e.g. race, highest educational degree). We used multivariable logistic regression to determine the relationship of sleep (dichotomized PSQI global score) with each psychological health outcome, first unadjusted, and then controlling for age, race, gender, years’ experience, highest educational degree, length of time employed in current position, hospital site, and service line. Next, we calculated adjusted odds ratios to determine the association between each PSQI sleep dimension score and dichotomized outcome variables (burnout, depression, anxiety, and compassion fatigue).

## Results

Across the three hospitals, 1400 registered nurses were sent a recruitment email. We received 535 unique responses (response rate 38%). The precise response rate was unclear, due to the nature of email recruitment and uncertainty with how many targeted individuals received the email as opposed to deletion, unavailability, not presently working, or medical leave. We evaluated the responses, and 112 surveys had greater than 80% missing data, and were subsequently removed from the analysis. We were left with 423 participants for final data analysis. Guided by principles of Tabachnick and Fidell [26], we assessed the amount of missing responses in the eligible responses. Previous evidence has demonstrated that upward of 20% missing data is common in psychological studies [27]. The percentage of missing data across individual items ranged from 0% to 1.4% for all variables except Habitual Sleep Efficiency PSQI subcomponent score (7.8%). We used case-wise deletion method and have noted the exact sample sizes (*N*) for each variable in Tables 3 and 4.

**Table 3.** Associations between Good Sleep Quality (PSQI ≤ 5) and work-related or psychological factors

	Unadjusted		Adjusted^†^	
	OR	95% CI	OR	95% CI
Burnout (*N* = 335)				
No symptoms	4.11	(2.37, 7.13)	5.65	(2.60, 12.27)^***^
Job satisfaction (*N* = 335)	0.72	(0.39, 1.33)	.939	(0.44, 5.59)
Depression screening (*N* = 304)				
PHQ2 < 3 (No depression)	6.70	(3.71, 12.10)^***^	6.91	(3.24, 14.72)^***^
Anxiety screening (*N* = 302)				
GAD-2 score < 3 (No anxiety)	4.79	(2.70, 8.48)^***^	10.75	(4.22, 27.42)^***^
Resilience (*N* = 299)				
BRS (High)	3.01	(1.49, 6.06)^**^	13.85	(2.91, 65.99)^***^
Compassion fatigue (*N* = 282)				
ComFat level <30 (low risk)	5.57	(2.26, 13.69)^***^	7.88	(1.97, 31.51)^***^

^†^Model adjusted for age, race, gender, years’ experience, highest educational degree, length of time employed in current position, hospital site, and service line. PHQ, Patient Health Questionnaire-2; GAD, Generalized Anxiety Disorder-2; BRS, Brief Resilience Scale; PSQI, Pittsburgh Sleep Quality Index; ComFat, Compassion Fatigue Self-test for Helpers;

^*^ *p* < .05,

^**^ *p* < .01,

^***^ *p* < .001; Following case-wise deletion, exact *N* used in models are reflected for each variable above.

**Table 4.** Associations between PSQI sleep dimensions and psychological health outcomes in frontline nursing workforce

PSQI sleep dimensions	Burnout	Depression	Anxiety	Compassion fatigue
	OR (95% CI)	OR (95% CI)	OR (95% CI)	OR (95% CI)
1: Subjective sleep quality (*n* = 347)	2.21 (1.41, 3.48) ^***^	0.66 (0.39, 1.11)	1.61 (0.92, 2.81)	1.81 (0.68, 4.83)
2: Sleep latency (*n* = 348)	1.09 (0.80, 1.48)	1.10 (0.76, 1.61)	0.87 (0.59, 1.28)	1.43 (0.74, 2.75)
3: Sleep duration (*n* = 348)	0.84 (0.59, 1.19)	0.88 (0.58, 1.33)	−0.88 (0.57, 1.37)	1.14 (0.53, 2.44)
4: Habitual sleep efficiency (*n* = 321)	0.97 (0.93, 1.02)	1.00 (0.95, 1.06)	0.979 (0.92, 1.04)	1.05 (0.93, 1.16)
5: Sleep disturbances (*n* = 348)	0.75 (0.45, 1.24)	0.75 (0.41, 1.37)	2.81 (1.42, 5.57) ^**^	0.890 (0.29, 2.78)
6: Use of medications (*n* = 347)	1.19 (0.93, 1.53)	0.90 (0.68, 1.20)	1.42 (1.01, 2.00) ^*^	1.32 (0.67, 2.60)
7: Daytime dysfunction (*n* = 343)	1.77 (1.26, 2.47) ^***^	0.21 (0.13, 0.33)^***^	2.86 (1.82, 4.49)^***^	2.32 (1.05, 5.11)^*^

OR, odds ratio. Adjusted for age, race, gender, years of experience, hospital site, and service line

^*^ *p* < .05,

^**^ *p* < .01,

^***^ *p* < .001; Following case-wise deletion, exact *N* used in models are reflected for each variable above.

The mean age of respondents was 42 years and predominantly female (87.5%). Half of participants reported being White (51.7%), 16% Hispanic, 16% Asian, and almost 11% Black. This sample represented a more diverse group compared to current national nursing workforce statistics [28]. The average number of years in primary position was 4 and more than one-quarter of nurses reported having a master’s degree or higher as their highest educational degree. The departments and service line where our participants delivered care primarily is listed in Table 1.

**Table 1.** Nursing sample demographics and characteristics

Characteristic	*N* (%)
Age (years)^†^	42 (12.34)
Race	
White	262 (51.7)
Hispanic	81 (16)
Asian	84 (16.6)
Black	55 (10.8)
Pacific islander	6 (1.2)
Gender	
Female	447 (87.5)
Martial status	
Married	268(52.4)
Length of time in primary position (years)^†^	4.34 (1.47)
Highest educational degree attained	
Bachelor’s degree	281 (67.9)
Master’s degree	104 (25.1)
Post-Master’s certificate	5 (1.2)
Doctor of Nursing Practice (DNP)	5 (1.2)
Doctor of Philosophy (PhD)	3 (0.7)
Primary service line/unit type	
Emergency department	30 (6.8)
Surgery/OR/PACU/Ambulatory surgery	70 (16)
Obstetrics/Postpartum/Nursery/NICU	53 (12.1)
Pediatrics/PICU	49 (11.2)
Cardiology (e.g. Interventional; Telemetry)	36 (8.2)
Oncology	24 (5.5)
Intensive care (adult)	70 (16)
Med/Surg	73 (16.7)
Average number of hours worked per week	
<30 h	31 (7.1)
31–40 h	317 (71.9)
>40 h	93 (21.1)

^†^ Mean (standard deviation).

Self-reported work-related and psychological outcomes of the nurses are presented in Table 2. More than half of participants reported burnout (64%) and almost 42% were dissatisfied with their job. One-third of participants reported intentions to leave their current job within a year. Sixty-seven percent of participants screened positive for depression (PHQ-2 ≥ 3) and more than three-quarters (77.9%) of the sample were positive for anxiety (GAD-2 score ≥ 3). Only 12% scored within the high category of resilience (brief resilience scale > 4.31). Ninety-two percent were positive for compassion fatigue (ComFat ≥ 30).

**Table 2.** Self-reported work-related and psychological outcomes of nurses

	*N* (%)
Burnout	
Symptoms present	245 (64)
Job satisfaction	
Dissatisfied with present job	160 (41.8)
Intention to leave current position in next year	123 (32.4)
Depression	
PHQ2 ≥ 3 (+ Depression)	195 (67.0)
Anxiety (GAD2)	
GAD-2 score ≥ 3 (+Anxiety)	225 (77.9)
Resilience	
BRS < 3 (Low)	67 (19.2)
BRS 3–4.30 (Medium)	240 (68.8)
BRS > 4.31 (High)	42 (12.0)
Sleep	
Self-reported sleep duration (hours)/night^†^	5.87 (1.33)
PSQI > 5 (Poor Sleep)	241 (80.9)
PSQI Global score^†^	9.27 (4.14)
Compassion fatigue	
ComFat level ≥ 30 (risk for compassion fatigue)	252 (92.0)

^†^Mean (standard deviation). PHQ, Patient Health Questionnaire-2; GAD, Generalized Anxiety Disorder-2; BRS, Brief Resilience Scale; PSQI, Pittsburgh Sleep Quality Index; ComFat, Compassion Fatigue Self-test for Helpers.

The majority of respondents (89.5%) reported less than 7 h of sleep per night over the past month, and almost 17% reporting less than 5 h per night. Almost all respondents (96%) reported the presence of sleep disturbances over the past month and 40% reported sleep disturbances at least weekly. More than half reported no use of sleep medication, while 18% reported using sleep medication three or more times per week. Almost 64% reported, on average over the past month, greater than 30 min to fall asleep (sleep latency) and more than half had habitual sleep efficiency scores less than 75%. Only 6% of respondents rated their subjective sleep quality as “very good” while half (50.2%) reported their sleep quality as “fairly poor” or “very poor.” Almost 40% of respondents reported experiencing daytime dysfunction at least weekly and 10% reported more than three times per week.

Table 3 shows the results of unadjusted and adjusted models that estimate the associations of good sleep (PSQI ≤ 5) with burnout, depression, anxiety, compassion fatigue, job satisfaction, and resilience. In the adjusted models, compared to participants who reported poor sleep (PSQI) > 5, those who reported good sleep (PSQI ≤ 5) had more than 5 times the odds of no burnout symptoms (OR: 5.65, 95% CI: 2.60, 12.27). Similarly, in adjusted models, those who reported good global sleep quality (PSQI ≤ 5) had increased odds of screening negative for depression (OR: 6.91, 95% CI: 3.24, 14.72), anxiety (OR: 10.75, 95% CI: 4.22, 27.42) and compassion fatigue (OR: 7.88, 95% CI: 1.97, 31.51). In both unadjusted and adjusted models, those who reported good global sleep quality (PSQI ≤ 5) also had significantly higher odds of high resilience scores (BRS > 3) (OR: 3.01, 95% CI: 1.49, 6.06 [unadjusted]; OR: 2.91, 95% CI: 2.91, 65.99 [adjusted]). Job satisfaction was not significantly associated with PSQI global sleep scores in either the unadjusted or adjusted models.

Table 4 shows the results of adjusted models that estimate the association of each PSQI sleep dimension score and burnout, depression, anxiety, and compassion fatigue. Subjective sleep quality was significantly associated with burnout (OR: 2.21, 95% CI: 1.41, 3.48), but sleep duration was not (OR: 0.84, 95% CI: 0.59, 1.19) (Table 4). Daytime dysfunction score was significantly associated with all four psychological outcomes: burnout (*p* < .001); depression (*p* < .001); anxiety (*p* < .001) and compassion fatigue (*p* < .05). More specifically, for every one-point increase in daytime dysfunction, there were 1.77 times higher odds of burnout and 2.86 times higher odds of anxiety. There were also significantly higher odds for anxiety with every one-point increase in the sleep disturbance score (OR: 2.81, 95% CI: 1.42, 5.57) or sleep medication dimension (OR: 1.42, CI: 1.01, 2.00).

## Discussion

This study aimed to understand the associations of sleep and its specific sleep dimension (e.g. latency, duration, and disturbances) with psychological health risks in nurses during the COVID-19 pandemic. We report here that overall poor sleep was associated with the presence of burnout, depression, anxiety, and compassion fatigue, as well as reduced job satisfaction and resilience. Different sleep dimensions were associated with psychological and work-related outcomes in nurses. Specifically, worse subjective sleep quality subcomponent scores were associated with odds of burnout, and higher daytime dysfunction scores were associated with all measures of psychological risk.

Our results indicate that levels of burnout, which were already at alarmingly high levels pre-pandemic, have been further increased during the COVID period. Burnout mitigation has increasingly been at the forefront of national initiatives to promote healthy work environments and subsequently strengthen US healthcare workforce. In 2018, the National Academy of Medicine formed the *Action Collaborative on Clinician Well-Being and Resilience* [29]. The intent of this collaborative was to promote awareness of the rising incidence of clinician burnout, prompt rapid evaluation of challenges to clinician well-being, and encourage investigation of multidisciplinary solutions that prevent adverse health outcomes such as stress, burnout, and suicidal behavior. In 2021, NAM also released their *Future of Nursing Report: 2020–2030* that dedicated two individual chapters toward the focus of mitigating burnout and adverse health outcomes in nurses and promoting healthy work environments [30]. Identifying central causes of stress and burnout in clinicians as well as targeted interventions to improve individualized efforts for personal health optimization, including effective sleep, should be at the forefront of workforce well-being research. More recently, the US Surgeon General released an advisory report calling for increased efforts to understand specific factors that precipitate or protect healthcare workforce from burnout [31]. This study contributes to the body of literature about the association of individual sleep dimensions and suboptimal psychological health outcomes in nurses, with particular attention to those working on the frontlines during the COVID-19 pandemic.

There are several implications for research, practice, and policy stemming from the new evidence presented in this paper. First, in addition to the evaluation of global sleep quality, our findings suggest that different sleep dimensions may yield varying impacts on different psychological outcomes. Researchers may replicate our methods and are encouraged to conduct analyses of individual PSQI sleep dimensions to interpret meaningful impact of targeted interventions on specific domains of sleep health for mental health outcomes. Next, this paper is among the early studies to investigate sleep specifically in the nursing workforce population working on the frontlines of the COVID-19 pandemic and notably through the assessment of isolated PSQI sleep dimensions. Nurses are the predominant HCW population in the United States with upward of 4 million nurses across the country [28]. Nurses often encounter physical, mental, emotional, and ethical challenges related to their job responsibilities and the clinical environments they work in. It remains unclear if our findings in this current study would be consistent across other clinical workforce disciplines and settings such as primary care. Future research should consider investigating individual sleep dimensions, as opposed to global sleep quality in other types of nurses, physicians, as well as other frontline workforce groups.

It also remains unclear how much the unprecedented changes in COVID-19 work environments either exacerbated or underscored associations among sleep dimensions, duration and quality, and adverse psychological health outcomes in this present study. Querstret et al. [32] published a scoping review in December 2020, which included studies published through 2018, and concluded that shift type and duration (e.g. night shift and longer shift times) as well as decreased recovery time between shifts were associated with poorer sleep and higher levels of fatigue in nurses. It can be assumed that these factors played some role in the associations found in our present study. All studies in the scoping review, however, were conducted prior to COVID-19 and may not be consistent with the associations we found at the height of a global pandemic. The scoping review also noted a variability of outcomes when comparing nursing-targeted interventions for sleep health. More research is recommended to test the mediating effect of nursing work environment characteristics (e.g. night shift, staffing ratios) between sleep interventions and nursing burnout or psychological outcomes. Furthermore, it is known that the circadian misalignment induced by working nonstandard shifts can also result in sleep disturbances [33]. Until we have more knowledge about the impact of work environment factors on sleep in nurses, we will not be able to differentiate which type of interventions (e.g. sleep hygiene, interventions to foster circadian adaptation to shift work [34], or changes in organizational workforce policies) shall be pursued. In lieu of this, our findings illuminate how sleep dimensions were potentially impacted at the height of a public health crisis and may be used to inform response efforts for nurse well-being in future similar events. Ongoing research about nurses in the immediate post-pandemic timeframe and beyond may help assess if the associations found in this study are sustained.

There are limitations to our study that should be considered when interpreting our findings. This study was cross sectional and therefore we cannot make conclusive statements about the directionality of relationships as well as the longitudinal impact of sleep behavior on psychological outcomes. The cross sectional survey design, based on the self-reported PSQI as opposed to objective assessments of sleep, is a limitation. For example, sleep duration PSQI dimension, based on self-report, was not associated with outcomes. On the other hand, high sleep disturbances, based on the PSQI dimension score as opposed to measures of frequent nocturnal awakenings or sleep fragmentation based on polysomnography or actigraphy, was associated with anxiety. Self-report of health in general poses some bias; including risk for recall bias (systematic errors based on a participant’s ability to accurately report recollections related to a given variable, sometimes reporting more or less despite the true value) or the Hawthorne effect (participants may modify their responses given the awareness that they are being observed). Future studies should incorporate objective assessment of sleep, coupled with subjective sleep quality and insomnia symptoms assessment. We further recommend increased health services research to understand the impact of nurse work environment factors on sleep health and potential risk for poor psychological health. Our sampling was also limited to one region and surveys may have introduced self-report bias. However, the demographics of our sample were aligned with the current national nursing workforce demographics that may potentiate the generalizability of our findings. More research is needed, specifically longitudinal design and objective assessment of sleep, that determines changes in sleep in the post pandemic period.

In summary, our study findings present evidence about the associations of sleep quality as well as PSQI sleep dimensions with nursing workforce psychological health and may be generalizable to other parts of the frontline health care workforce. Poor sleep quality was common and associated with increased burnout in nurses. Sleep quality appeared to be more strongly related to burnout than sleep duration. However, future research should test the mediating impact of nursing work environment factors such as shift type and duration. Our findings suggest that ongoing research should be done to test and develop sleep improvement programs in nurses to determine their impact on psychological outcomes in clinicians.

## Data Availability

Available upon reasonable request.
